# Remote ischaemic conditioning: cardiac protection from afar

**DOI:** 10.1111/anae.12973

**Published:** 2015-02-26

**Authors:** V. Sivaraman, J. M. J. Pickard, D. J. Hausenloy

**Affiliations:** ^1^ The Hatter Cardiovascular InstituteUniversity College LondonLondonUK

## Abstract

For patients with ischaemic heart disease, remote ischaemic conditioning may offer an innovative, non‐invasive and virtually cost‐free therapy for protecting the myocardium against the detrimental effects of acute ischaemia‐reperfusion injury, preserving cardiac function and improving clinical outcomes. The intriguing phenomenon of remote ischaemic conditioning was first discovered over 20 years ago, when it was shown that the heart could be rendered resistant to acute ischaemia‐reperfusion injury by applying one or more cycles of brief ischaemia and reperfusion to an organ or tissue away from the heart – initially termed ‘cardioprotection at a distance’. Subsequent pre‐clinical and then clinical studies made the important discovery that remote ischaemic conditioning could be elicited non‐invasively, by inducing brief ischaemia and reperfusion to the upper or lower limb using a cuff. The actual mechanism underlying remote ischaemic conditioning cardioprotection remains unclear, although a neuro‐hormonal pathway has been implicated. Since its initial discovery in 1993, the first proof‐of‐concept clinical studies of remote ischaemic conditioning followed in 2006, and now multicentre clinical outcome studies are underway. In this review article, we explore the potential mechanisms underlying this academic curiosity, and assess the success of its application in the clinical setting.

## Introduction

Cardiovascular disease remains the leading cause of death and disability worldwide, accounting for 17 million deaths in 2008, of which ischaemic heart disease (IHD) is a major contributor 1. For emergency IHD patients presenting with an acute myocardial infarction (MI), the treatment of choice is reperfusion of the acutely ischaemic myocardium using percutaneous coronary intervention (PCI). For stable IHD patients with multivessel coronary artery disease, the treatment of choice is usually revascularisation by coronary artery bypass graft (CABG) surgery. In both clinical settings, the heart is subjected to the detrimental effects of acute ischaemia‐reperfusion injury (IRI), the result of which is cardiomyocyte death, impaired cardiac contractile function, risk of onset of heart failure and death. Despite advances in treatment, the morbidity and mortality in these patients remains significant. Specifically, the risk profile of patients undergoing CABG surgery has increased 2, 3. There exists a need therefore to research and develop techniques that will lower mortality further, and one such target is to reduce myocardial reperfusion injury 4. This is a paradoxical phenomenon of myocardial damage that occurs on return of blood supply to the ischaemic myocardium 5. The exact contribution of reperfusion injury to the overall infarct size has been difficult to quantify, but it has been estimated to be in the region of 50% of the final infarct size 6. Therefore, novel therapeutic interventions are required to protect the heart against the detrimental effects of acute IRI.

In this review article, we discuss the non‐invasive, virtually cost‐free strategy of remote ischaemic conditioning (RIC), which has made the leap from being an academic curiosity to a potential clinical therapy.

## Methods

We undertook a PubMed search using the keywords myocardial infarction, reperfusion, ischaemia, remote, preconditioning, postconditioning, perconditioning, cardiac and vascular surgery, CABG, coronary intervention, and cardiopulmonary bypass and reviewed papers retrieved using these search terms. In addition, references to papers were examined, and papers of relevance were also reviewed. Certain landmark papers have been referred to, independent of the PubMed search criteria.

## RIC: history and evolution

The endogenous cardioprotective phenomena of ischaemic preconditioning, first discovered in 1986 7, and ischaemic postconditioning, later described in 2003 8, have the ability to protect the heart against acute lethal IRI, and are reviewed elsewhere in this journal 9, 10. The major disadvantage of ischaemic preconditioning and ischaemic postconditioning, however, is the requirement to apply an invasive intervention directly to the heart. In this regard, the phenomenon of RIC, which allows the protective stimulus to be applied to an organ or tissue away from the heart, has the advantage.

The first experimental study to describe this cardioprotective phenomenon was published by Przyklenk et al. in 1993 11. In a canine heart model, applying 45 min cycles of occlusion and reflow to the circumflex coronary artery reduced infarct size induced by 45 min occlusion and 3 h reperfusion of the left anterior descending artery, suggesting that cardioprotection could be transferred from one coronary territory to another. This transfer of cardioprotection was then extended to a remote organ, the kidney, by McClanahan et al., who demonstrated that 10 min of occlusion and reflow in the renal artery could reduce infarct size induced by 30 min ligation and 3 h reperfusion of the left main coronary artery, extending the paradigm from intra‐cardiac to inter‐organ protection 12. Subsequent experimental studies have reported that the preconditioning stimulus could be applied to a number of different organs remote from the heart, including the intestine, liver and brain. However, in terms of facilitating the translation of RIC into the clinical setting, the major advance was made by Birnbaum et al., who discovered that the RIC stimulus could be applied to the gastrocnemius muscle of the hindlimb, by partially occluding the femoral artery 13. Furthermore, Oxman et al. demonstrated that the conditioning stimulus could be applied non‐invasively, using a tourniquet applied to the hindlimb 14. The discovery that RIC could be elicited non‐invasively, by simply inflating and deflating a cuff placed on the upper arm or leg in human volunteers, greatly facilitated its translation into the clinical setting (see below).

One other major advantage of RIC is its ability to confer cardioprotection when applied at a number of different time points in relation to the index myocardial ischaemia‐reperfusion episode, a feature that has also facilitated its clinical application. The early experimental studies focused on applying the conditioning stimulus immediately before the index myocardial ischaemic episode (remote ischaemic *pre*conditioning), with subsequent experimental studies reporting efficacy with the conditioning stimulus applied at varying time points. These include 12–24 h before the index myocardial ischaemic episode (delayed remote ischaemic *pre*conditioning); after the onset of myocardial ischaemia, but before reperfusion (remote ischaemic *per*conditioning); at the onset of myocardial reperfusion (remote ischaemic *post*conditioning); and most recently, even after 15 min of myocardial reperfusion has elapsed (delayed remote ischaemic *post*conditioning).

### Mechanisms underlying RIC

**Figure 1:**
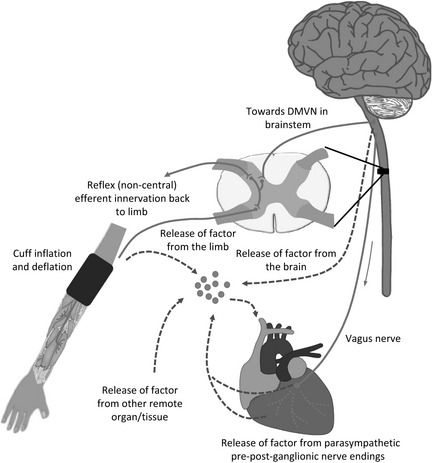
Hypothetical model highlighting potential mechanistic pathways underlying remote ischaemic conditioning. It has been proposed that brief non‐lethal periods of ischaemia‐reperfusion applied to the upper or lower limb, using serial inflation/deflations of a cuff, causes the release of local autocoids (such as adenosine, bradykinin, opioids) which then activate sensory afferent neurones in the upper or lower limb that in turn relay the cardioprotective signal to the dorsal motor vagal nucleus (DMVN) in the brainstem. At this point, it is not clear how the cardioprotective signal reaches the heart or other organs. Several hypotheses have been suggested. (1) Activation of nuclei within the DMVN results in increased vagal nerve firing to the heart, which via the release of acetylcholine (Ach), and subsequent activation of muscarinic Ach receptors, induces the cardioprotective phenotype. (2) It is largely agreed that a blood‐borne dialysable cardioprotective factor is released into the systemic circulation, from where it conveys the cardioprotective effect to the heart and other organs. However, the actual source of the blood‐borne cardioprotective factor is not clear, although possibilities include: (i) the conditioned limb itself; (ii) the central nervous system, possibly the brainstem; (iii) pre‐/postganglionic parasympathetic nerve endings within the heart; or (iv) a non‐conditioned remote organ/tissue.

The actual mechanistic pathway underlying RIC‐induced cardioprotection remains unclear, but it can be divided into three interrelated events: (i) generation of the cardioprotective signal in the conditioned remote organ or tissue; (ii) the pathway that conveys the cardioprotective signal from the conditioned remote organ or tissue to the heart; and (iii) the activation of intracellular signalling pathways within the heart that mediate the cardioprotective effect (Fig. 1). The current paradigm dictates there are both neural and humoral aspects to (i) and (ii), and their involvement and potential interdependence are discussed below. The myocardial signalling pathways are thought to be similar to those that are recruited in ischaemic preconditioning.

A number of experimental studies have implicated a blood‐borne factor(s) generated in the RIC‐treated animal as potential mediators of cardioprotection. The evidence for this is derived from the following studies. Gho et al. noted that RIC would only protect the heart if the conditioned intestine was reperfused, suggesting that reperfusion was required to wash the cardioprotective factor out of the conditioned tissue 15. Dickson et al. 16, 17 discovered that the ischaemic preconditioning effect could be transferred from one rabbit to another non‐preconditioned rabbit, first via whole blood transfusion, and second via transfer of coronary effluent (IPC effluent) from a preconditioned, to a naïve isolated heart. Hence, the presence and necessity of a blood‐borne cardioprotective factor is clear. This model has been reproduced across several species 18, 19, 20, and has played a central role in recent characterisation of the humoral factor. Preliminary evidence suggested that an intact opioid receptor system was required for protection to occur 19, 21; however, opioid levels were not raised in IPC effluent 21. A subsequent study demonstrated raised adenosine levels in IPC effluent and, via use of the adenosine receptor blocker, 8‐(‐p‐sulfophenyl) theophylline (8‐SPT), that adenosine receptor activation was required for effluent‐mediated protection 22. Combined together, this evidence could suggest adenosine and opioid receptor cross‐talk 23, 24.

Current evidence suggests the factor is between 3.5 and 30 kDa, thermolabile and hydrophobic. Using dialysis membranes to fractionate the proteins within coronary effluent or serum according to their molecular weight, several studies demonstrated the factor to be smaller than 30 kDa 19, 20 and, crucially, larger than 3.5 kDa 18. This would suggest that small molecules, such as adenosine and opioids, are not essential for RIC (adenosine 267.24 Da, opioids 500–800 Da, bradykinin 1060.22 Da). Moreover, using a series of chromatographic and heating steps, it was proposed that the factor was both thermolabile and hydrophobic, indicating it may be a protein 18, 20. Proteomic analysis of plasma, via a combination of two‐dimensional gel electrophoresis and mass spectrometry, unmasked an altered plasma proteome following RIC. Studies by Lang et al., Hepponstall et al. and Hibert et al. found 4, 51 and 30 differentially expressed proteins, respectively, in RIC plasma relative to control 25, 26, 27. These proteins, linked to the regulation of various cellular functions, including the acute phase response, immune response, haemostasis and lipid transport, suggested a complex interaction of signalling pathways in response to RIC. Broad proteomic analyses, however, are not well suited to the hypothesis of a single, small, low‐abundance protein conveying the cardioprotection, because of the large number of high‐abundance proteins in plasma. With this in mind, two studies have analysed coronary effluent using liquid chromatography and mass spectrometry, the advantage being that the majority of plasma proteins (high‐abundance) are removed 22, 28. Initial investigation revealed 185 unique proteins in coronary effluent, following five cycles of 5 min global ischaemia, and 11 min reperfusion in an isolated rat heart. Only 30.3% were plasma proteins, with the remainder originating from the cytoplasm and various intracellular organelles. A subsequent study alluded in the discussion to a proteomic (liquid chromatography/mass spectrometry) analysis of coronary effluent from isolated rat hearts, which found that 8–10 peptides were increased by > 50% relative to control effluent, with in the order of 100 uncharacterised ions corresponding to metabolites or proteins 22. Thus, although high‐abundance proteins are largely excluded in coronary effluent, a significant amount of protein is still released, indicating that effluent must be further fractionated before mass spectrometry, if a potential biomarker is to be found.

Several experimental studies have suggested that the cardioprotective effect elicited by a RIC stimulus delivered in vivo was still present, even when the heart was isolated and subjected to acute MI either on a Langendorff apparatus (isolated perfused hearts) 29, or in a transplanted recipient animal 30. These findings suggest that an intact neural pathway to the heart was not required for RIC cardioprotection during the acute MI, but it does not of course exclude the need for an intact neural pathway at the time of the RIC stimulus.

Although the identity of the blood‐borne cardioprotective mediator of RIC remains unknown, a number of candidate molecules have been suggested including opioid 21, adenosine 22, bradykinin 31, erythropoietin, calcitonin gene related peptide, stromal derived factor 1‐alpha (SDF1‐α) 32, hypoxia inducible factor 1‐alpha (HIF1‐α) and nanoparticles produced by cells called exosomes 33.

A number of experimental studies have suggested the involvement of a neural pathway in RIC cardioprotection. The evidence for this is summarised below. Gho et al. first noted that the ganglion blocker, hexamethonium, blocked RIC‐induced cardioprotection elicited by intestinal conditioning, thereby implicating a role for the autonomic ganglia of the sympathetic and parasympathetic nervous systems in inter‐organ protection 15. A subsequent study in human volunteers confirmed that trimethaphan, an autonomic ganglionic blocker, abrogated limb RIC protection of endothelial function 34. Two studies, however, did not show any abolition of RIC with hexamethonium 35, 36, suggesting that further investigation is perhaps necessary.

Given the involvement of the autonomic nervous system in RIC, it is logical to hypothesise that the RIC cardioprotection is dependent on an intact sensory afferent neuronal pathway at the remote organ or tissue. Indeed, transection of the femoral nerve before application of the RIC stimulus abolishes cardioprotection 37, 38. Moreover, Ding et al. noted that brief renal artery occlusion resulted in increased afferent renal nerve activity, and nerve transection also abolished RIC‐induced cardioprotection 39. Similarly, direct stimulation of the sensory nerve of the remote organ or tissue has been reported to reproduce the cardioprotective effect elicited by RIC 40, 41, 42. Finally, stimulation of cutaneous sensory nerves, using either topical application of capsaicin 43 or surgical skin incision (see later section on remote preconditioning of trauma) 44, 45, has been reported also to mimic RIC cardioprotection.

The question arose, therefore, of how the brief ischaemic burden to the tissue or organ is translated to sensory afferent activation. Experimental studies have demonstrated that the application of brief ischaemia–reperfusion to a remote organ or tissue generates factors such as adenosine 39, 46, 47, bradykinin 31 and calcitonin gene related peptide 48 in the remote organ or tissue, which then stimulate the local sensory neural afferent pathway. Indeed, the adenosine receptor antagonist 8‐SPT abrogated the increased afferent renal nerve activity observed by Ding et al. following brief renal artery occlusion 39. In addition, intra‐arterial injection of adenosine, while not at a level sufficient to elicit cardioprotection alone, was able to induce cardioprotection via a mechanism requiring an intact sciatic nerve 38. Thus, it seems RIC induces local release of a neuro‐active factor that in turn activates sensory afferent neurones, and initiates the cardioprotective message.

The final element concerns the efferent limb of the neural pathway to the heart. Initial studies demonstrated that vagal nerve stimulation 49, 50 mimicked the cardioprotective effect of RIC, whereas with bilateral vagotomy this was abrogated 49, 50. An elegant study by Mastitskaya et al. demonstrated, using optogenetics, that activation of the dorsal motor nucleus of the vagus nerve was sufficient to induce cardioprotection 51. Moreover, this effect was abrogated in the presence of atropine. Two papers, however, question this paradigm; first, spinal cord transection at C7, by removing central nervous innervation, did not abolish the remote preconditioning of trauma 52. Second, there is evidence that vagal nerve stimulation before index ischaemia increases infarct size 53.

Initial experimental studies had implicated a blood‐borne cardioprotective pathway, and an intact neural pathway to the conditioned organ or tissue. Recent studies have investigated the interaction between these two signalling components.

It has been reported that RIC by limb ischaemia or intra‐arterial adenosine releases a dialysable blood‐borne cardioprotective factor(s), the release of which required intact sensory innervation of the limb and was blocked by pre‐treatment with the nitric oxide (NO) donor, S‐nitroso‐N‐acetylpenicillamine 38. Interestingly, production of NO, via activation of NO synthase, is seemingly not required for production of the cardioprotective factor, but is required for induction of the protective phenotype 54.

An important experimental study by Redington et al. demonstrated that either direct femoral nerve stimulation or topical application of capsaicin generated a dialysate that was able to reduce MI size in a naïve isolated rabbit heart, providing the first evidence that the neural pathway to the conditioned limb was required to generate the blood‐borne cardioprotective factor 43.

In an interesting clinical study, Jensen et al. found that plasma dialysate obtained from RIC‐treated diabetic patients with sensory neuropathy failed to limit MI size in an isolated naïve rabbit heart, although plasma dialysate harvested from RIC‐treated non‐diabetic and diabetic patients without sensory neuropathy was cardioprotective 55. Therefore, the current paradigm suggests that the conditioned limb generates adenosine in the local circulation, which then activates the sensory neural pathway (through a mechanism dependant on NO), leading to the activation of dorsal nuclei within the brainstem. How the cardioprotective signal is then conveyed from the brainstem to the heart remains unclear. The current evidence suggests the presence of both vagal and humoral influences 43, 51.

The concept of remote preconditioning of trauma originally arose from the observation that patients who had undergone abdominal aortic aneurysm (AAA) surgery had a greater risk for MI, a greater infarct size and increased mortality compared with those who had not undergone surgery. Indeed, Ren et al. observed that infarct size in mice increased with prior remote vascular surgery 44. However, they subsequently demonstrated that a small cutaneous incision to the abdomen was able to limit MI size, in a manner similar to RIC. Two further studies have described such a phenomenon, with the current paradigm suggesting the protection occurs via activation of Aδ‐ and C‐sensory afferent neurons 45, 52, which confer protection to the heart, perhaps independent of central innervation.

There is some evidence, although inconsistent, that a systemic inflammatory response is obtained in the setting of reperfusion injury. Reperfusion injury per se is associated with a neutrophilic infiltration within the target organ 4. Remote ischaemic conditioning seems to have an anti‐inflammatory effect. For instance, a group conducting experiment on skeletal muscle flaps noted that RIC reduced the number of leucocytes adhering the postcapillary venules 56. Similarly, RIC decreased overall levels of interleukin‐1β and interleukin‐6 in a porcine model of lung IRI. In a human forearm endothelial model of IRI, neutrophil adhesion was reduced by RIC, as was phagocytosis 57. Even pro‐inflammatory gene expression in leucocytes appears to be suppressed, while anti‐inflammtory gene expression is up‐regulated 58. However, when a panel of circulating cytokines was analysed in adults and children undergoing cardiac surgery, RIC did not produce any changes in anti‐inflammatory or pro‐inflammatory cytokines 61, 62. In a similar study by Albrecht et al., circulating levels of interleukin‐1β, interleukin‐8 and tumour necrosis factor‐α were increased in patients subjected to RIC before cardiopulmonary bypass, compared with controls 61. This pro‐inflammatory response directly contradicts pre‐clinical evidence and hence, the exact role of a systemic response is unclear and needs further investigation.

## Clinical translation of RIC

The first major step to the clinical translation of RIC was the discovery that the RIC stimulus could be delivered non‐invasively, by simply applying cycles of inflation–deflation using a standard blood pressure cuff placed on the upper arm 62, 63. The first study to investigate the effect of limb RIC protection of the heart, using this non‐invasive intervention, was a small proof‐of‐concept clinical study published in 2000, comprising eight patients undergoing CABG surgery. The authors did not find any cardioprotection in patients randomly assigned to the limb RIC protocol (two cycles of 3 min upper arm cuff inflation, and 2 min deflation). Subsequently, Kharbanda et al. demonstrated in human volunteers that limb RIC (three cycles of 5 min cuff inflation–deflation) protected the contralateral limb against ischaemia‐induced endothelial dysfunction, as assessed by flow‐mediated dilatation 63. This important study established the standard upper limb RIC protocol used in a number of subsequent clinical studies.

Remote ischaemic conditioning has been investigated in a number of different clinical settings of acute myocardial IRI:

1. CABG surgery, in which the heart is subjected to global ischaemic injury as the heart goes onto cardiopulmonary bypass (CPB), followed by global reperfusion injury as the heart comes off CPB. The global acute IRI that results can be detected as peri‐operative myocardial injury by measuring a rise in serum cardiac enzymes such as creatinine kinase (CK‐MB) and troponin T and I. It can also be detected as areas of late gadolinium enhancement (which corresponds to the area of myocardial necrosis) on cardiac magnetic resonance imaging;
2. Major non‐cardiac vascular surgery, in which peri‐operative myocardial injury measured by a rise in serum cardiac enzymes such as CK‐MB and troponin T and I occurs in 20–30% of patients;
3. Elective PCI. One component of the peri‐procedural myocardial injury that occurs during PCI is due to regional ischaemic injury and coronary microembolisation, the extent of which can be measured by the rise in serum cardiac enzymes;
4. ST‐segment elevation MI (STEMI) treated by primary PCI. Patients presenting with an acute STEMI will usually have a major thrombotic occlusion in one of the major epicardial coronary arteries, resulting in acute myocardial ischaemia, the treatment for which is timely reperfusion using primary PCI. The price for restoring blood flow in the infarct‐related artery, however, is further myocardial injury and cardiomyocyte death – termed myocardial reperfusion injury 4.

## RIC in cardiac surgery

A protocol of four cycles of 5 min ischaemia‐reperfusion of the forearm was applied to children before congenital heart surgery 60. The group that had the RIC protocol had lower peri‐operative myocardial injury, as determined by postoperative troponin I, lower inotropic requirements at 3 and 6 h, and lower airway pressures at 6 h postoperatively. In the adult cardiac surgical setting, our group was the first to provide evidence that RIC could be effective 64. Remote ischaemic conditioning was applied after the induction of anaesthesia, in the form of three cycles of 5 min ischaemia‐reperfusion of the forearm. In the 27 patients in the RIC group, there was a 43% reduction in peri‐operative myocardial injury, as determined by troponin levels over 72 h, compared with the control group. Similar studies in the cardiac surgical setting have followed, and have confirmed the protective properties of RIC. For instance, a recent trial by Thielmann et al. randomly assigned 329 elective CABG patients to either RIC or a sham protocol. The troponin release was not only lower in the RIC group, but this group also had a lower all‐cause mortality when assessed after 1.54 years 65. Table 1 gives a brief overview of the important clinical trials conducted in cardiac surgical patients.

**Table 1:** Some key clinical trials of remote ischaemic conditioning (RIC) in cardiac surgery

Study	n	Details of surgery	Details of anaesthesia	Study comments	Results
Cheung et al. 60	37	Paediatric congenital cardiac surgery (blood cardioplegia)	I – sevoflurane M – isoflurane + air + O_2_	Single‐blinded Elective patients with no co‐morbidities	↓ TnI ↓ Inotrope score at 3 and 6 h ↓ Airway resistance at 6 h
Hausenloy et al. 64	57	Elective adult CABG surgery (cold blood cardioplegia and intermittent cross‐clamp fibrillation)	I – midazolam + propofol/etomidate + fentanyl M – propofol TCI	Single‐blinded Elective patients with no co‐morbidities	↓ AUC of TnT (43%)
Venugopal et al. 66	45	Elective adult CABG surgery ± valve (cold blood cardioplegia only)	I – midazolam + propofol/etomidate + fentanyl M – isoflurane + air + O_2_ or propofol TCI	Single‐blinded Elective patients with no co‐morbidities Diabetes were not studied	↓ AUC of TnT (42.2%)
Thielmann et al. 67	53	Elective CABG with cold crystalloid cardioplegia	I – sufentanil + etomidate M – isoflurane or propofol	Single‐blinded Elective patients with no co‐morbidities	↓ AUC of TnI (44.5%)
Hong et al. 68	130	Elective off pump CABG	I – midazolam + sufentanil M – sevoflurane + remifentanil	Single‐blinded RIC induced after knife to skin	↓ AUC of TnI (26%) which was not significant
Rahman et al. 69	162	Elective and urgent CABG with cold blood cardioplegia	I – etomidate + fentanyl M – propofol with isoflurane or enflurane during bypass	Double‐blinded Well designed with multiple secondary endpoints	No significant difference in Tn, inotrope score, ventilator requirements, kidney injury
Li et al. 70	81	Elective aortic valve replacement with cold blood cardioplegia	I – midazolam M – fentanyl + propofol and intermittent isoflurane	Single‐blinded Elective patients with no co‐morbidities Preconditioning and per conditioning examined	↓ in TnI with RIPerC only at 5 min before and 30 min after cross‐clamp removal
Wagner et al. 71	120	Elective CABG ± valve with cold crystalloid cardioplegia	I – sufentanil + diazepam M ‐ sufentanil + diazepam	Single‐blinded RIC applied 18 h before surgery ↑ TnI with tramadol	↓ in TnI only at 8 h
Karuppasamy et al. 59	104	Elective CABG ± valve with cold blood cardioplegia	I – midazolam + remifentanil + propofol M – isoflurane before bypass, propofol on and after bypass	Single‐blinded Strict anaesthetic regimen on isoflurane pre‐bypass No isoflurane at reperfusion	No change in troponin, CK‐MB or BNP No change in inflammatory cytokines
Wu et al. 72	75	Elective mitral valve surgery with blood cardioplegia	Premedication: scopolamine + diazepam I – midazolam + fentanyl M – midazolam + sufentanil	Two protocols of RIC tested. RIC 1 was 3× arm. RIC 2 involved 3× arm and 2× leg	↓ AUC of TnI
Lucchinetti et al. 73	55	Elective CABG with cold blood cardioplegia	I – propofol + fentanyl/sufentanil/remifentanil M – isoflurane + opioids	RIC started along with isoflurane at the same time Isoflurane throughout surgery	No change in TnT or BNP No change in inflammatory reponse
Young et al. 74	96	Double or triple valve, CABG + valve or isolated mitral valve, redo CABG, isolated CABG with < 50% ejection fraction all with warm blood cardioplegia	Premedication: zopiclone + midazolam I – midazolam + fentanyl M – propofol + isoflurane + fentanyl top‐ups	Double‐blinded RIC applied at the time of surgical incision	↓ TnT at 6 h and 12 h in RIC group after secondary analyses to adjust for sulphonylurea and statin use, cross‐clamp and bypass time, Euroscore. No change in AKI ↑ inotrope requirement in RIC group
Kottenberg et al. 75	72	Elective CABG surgery with cold crystalloid cardioplegia	I – sufentanil + etomidate M – two groups – one isoflurane and one propofol TIVA	Single‐blinded Four‐arm trial to examine the effects of anaesthesia	↓ AUC of TnI (50%) only in the isoflurane group
Hong et al. 76	1280	All elective cardiac surgery both off pump and on pump with cold blood cardioplegia	I – midazolam + etomidate + sufentanil M – propofol + remifentanil	Multicentre trial Study combined RIPreC with RIPostC Primary endpoint was composite outcome	No difference in primary outcome No difference in ICU/hospital stay
Thielmann et al. 77	329	Elective CABG surgery with cold crystalloid cardioplegia	I – sufentanil + etomidate M – isoflurane or propofol	Single‐centre Double‐blinded Mortality was taken as a safety endpoint Propofol used in only 79 patients	↓ AUC of TnI (17.3%) Lower all cause mortality at 1 year Lower MACCEat 1 year

I/M, induction/maintenance; Tn, troponin; CABG, coronary artery bypass graft; TCI, target‐controlled infusion; AUC, area under the curve; RIPerC, remote ischaemic perconditioning; CK‐MB, creatinine kinase MB isoenzyme; B‐type natriuretic peptide; AKI, acute kidney injury; TIVA, total intravenous anaesthesia; RIPreC/RIPostC, remote ischaemic pre/postconditioning; MACCE, major adverse cardiovascular and cerebral events

The biggest limitations of the clinical trials in the cardiac surgical setting lie in their study design. The vast majority of them have been single‐centre trials examining small groups of patients admitted for elective CABG surgery. This represents fewer than 50% of patients undergoing major cardiac surgery in England and Wales 78. Moreover, most of the trials have been single‐blinded, leaving room for bias. In fact, one meta‐analysis suggests that single‐blinded trials were mostly positive, while double‐blinded ones were mostly negative 79. Second, the primary endpoint used in almost all the trials was the area under the curve of a 48‐h or 72‐h collection of troponin or CK‐MB postprocedure. While there is evidence that higher troponin and CK‐MB levels after cardiac surgery are associated with increased mortality at one year, much of this evidence comes from small, single‐centre trials, with a mixture of prospective, retrospective and post‐hoc analyses, as discussed in a meta‐analysis by Petaja et al. 80. This represents only a surrogate endpoint, and stronger clinical endpoints such as postoperative morbidity and mortality, and patient‐centred indices such as quality of life, are lacking. Third, many of the initial studies excluded diabetic patients, as they were proof‐of‐concept trials. In fact, complex high‐risk patients, with unstable angina, recent MI, renal disease and complex surgery, were not studied. Given that the cardiac surgical casemix is gradually increasing in risk profile, this represents another major drawback.

Two large multicentre, double‐blinded randomised controlled trials aim to address these limitations. The ERICCA (Effect of Remote Ischaemic preConditioning on clinical outcomes in patients undergoing CABG surgery) trial, run by our group, is currently recruiting at 30 centres in the UK, and aims to recruit 1610 high‐risk patients undergoing CABG, with or without valve surgery. The primary endpoints in this trial are cardiovascular death, non‐fatal MI, coronary revascularisation and stroke at one year, with various secondary endpoints, including quality of life and exercise tolerance 81. The preliminary results of this trial should be available late next year. The RIPHeart (Remote Ischaemic Preconditioning for Heart surgery) study aims to recruit all patient undergoing any form of surgery involving CPB 82, with primary endpoints similar to those of ERICCA.

One of the first major trials to give a negative result was conducted by Bonser's group 69. This was a well‐designed, double‐blinded, placebo‐controlled trial that went to great lengths to ensure blinding. They randomly allocated 162 patients to either a control group or an RIC group, with a protocol of three cycles of 5 min ischaemia‐reperfusion of the forearm. Only elective or urgent, non‐diabetic CABG patients, with no history of unstable angina in the past 48 h were included. Remote ischaemic conditioning did not result in a reduction in peri‐operative myocardial injury, as determined by 48‐h area under the curve of troponin. Since then, there have been other studies that were negative (see Table 1), and there are possibly two major reasons for this.

First, the translation of RIC into the clinical setting is not straightforward. Remote ischaemic conditioning in the pre‐clinical setting was investigated in young animals that underwent myocardial IRI by direct ligature applied to the coronary artery. There was no pre‐existing disease state, and almost none of the models included any form of cardiopulmonary bypass. Direct translation into the clinical setting of this model is not feasible, as many of our patients have co‐existing morbidity. For instance, unstable angina, which is characterised by intermittent ischaemia, is a form of preconditioning, and patients with unstable angina who then have a MI often do better than patients whose first presentation is an MI 83, 84. Whether RIC would work over and above an already preconditioned heart is unclear. In addition, patient with diabetes are harder to precondition due to various factors 85. Furthermore, cardiopulmonary bypass itself is now an innovative technology that involves mild hypothermia and cardioplegia, all of which are protective for the heart 86. Thus, while the early positive trials included elective patients with few co‐morbidities 60, 64, Bonser and co‐workers included both elective and urgent patients with previous acute coronary syndromes. Second, the importance of concomitant medication cannot be underestimated. During the early years of ischaemic preconditioning (IPC) research, several pharmacological agents were found to mimic IPC. Some of these agents included volatile anaesthetic agents, glyceryl trintitrate, opioids such as remifentanil, fentanyl and morphine, insulin, atorvastatin, nicorandil, abciximab, clopidogrel and cangrelor 87. Patients referred for cardiac surgery are often on one or more of these drugs, or may be administered these drugs during the course of their surgery. It may be that patients might already have been protected, and the RIC stimulus did not confer any further benefit. However, the question remains whether RIC might further add to this protection, by increasing either the intensity or the timing of the stimulus. In fact, our group is currently recruiting patients for a four‐arm, double‐blinded, randomised placebo‐controlled trial to understand the effects of RIC and glyceryl trinitrate (the ERIC‐GTN study (www.clinicaltrials.gov, NCT01864252).

## RIC in major non‐cardiac vascular surgery

Ali et al. were the first to publish on the phenomenon of RIC in the setting of major vascular surgery 88. Patients having elective open AAA repair were randomly assigned to have a protocol of RIC that involved two epsiodes of 10 min ischaemia followed by reperfusion. This was achieved by cross‐clamping the iliac artery. Both groups were well‐matched, and RIC reduced myocardial injury, infarction and renal impairment significantly in these patients. However, in three small proof‐of‐concept trials by Walsh and colleagues, RIC in various vascular surgical settings gave mixed results. In the setting of endovascular aneurysm repair, RIC did not result in a reduction in cardiac injury, but there was a reduction in the urinary biomarker of renal injury. On the other hand, patients undergoing carotid endarterectomy and elective open AAA repair did not experience any benefit from an RIC protocol in terms of myocardial, renal or neurological injury. All of these trials were small proof‐of‐concept trials and large‐scale studies are yet to be performed in the setting of vascular surgery.

## RIC in elective PCI

An early study by Iliodromitis et al. did not show any evidence of benefit from an RIC protocol administered in patients undergoing elective PCI. However, this study may have been underpowered 89. The first clinical study to demonstrate the cardioprotective effects of RIC in stable IHD patients undergoing coronary revascularisation by elective PCI was by Hoole et al. 90. In the CRISP trial, where 202 patients were randomly assigned to either RIC or control, RIC reduced troponin release at 24 h following elective PCI, suggesting lower levels of periprocedural injury. Furthermore, the RIC group had a lower incidence of chest pain postoperatively, and fewer major adverse cardiovascular and cerebrovascular events (MACCE) at six months. In a follow‐up of 195 patients six years later, the RIC group continued to have lower rates of MACCE compared with the control group 91. Since then, a number of clinical studies investigating the protective effect of RIC have demonstrated mixed results in the elective PCI setting (Table 2).

**Table 2:** Some key clinical trials of remote ischaemic conditioning (RIC) in elective percutaneous coronary intervention (PCI)

Study	n	Study comments	Results
Illiodromitis et al. 89	41	Single‐centre unblinded study RIC performed in the catheter lab Single‐vessel disease	↑ TnI and CK‐MB release in the RIC group after 48 h
Hoole et al. 90	202	Single‐centre study Single‐blinded Blinded follow‐up for MACCE RIC performed 2 h before procedure Both single and combined targets stented	↓ TnI at 24 h after PCI in RIC group ↓ Chest discomfort and ST segment deviation in RIC group ↓ MACCE at 6 months in RIC group
Ghaemian et al. 92	80	Single‐centre unblinded study RIC stimulus applied to thigh 1 h before procedure Patients with previous MI were included	↓ TnI at both 12 and 24 h RIC determined to independently predict reduced periprocedural injury
Luo et al. 93	205	Single‐centre unblinded study Both single and combined targets stented	↓ TnI at 16 h in RIC group No change in renal outcome
Carrasco‐Chinchilla et al. 94	232	Single‐centre single‐blinded study Remote postconditioning protocol with stimulus applied 5 min after the balloon inflation/stent deployment	No difference in TnI at 24 h between groups Diabetic patients in the RIC group had a larger incidence of PCI‐MI

Tn, troponin; CK‐MB, creatinine kinase MB isoenzyme; MACCE, major adverse cardiovascular and cerebral events; MI, myocardial infarction.

## RIC in STEMI patients treated by Primary PCI

It is important to note that an RIC intervention in the setting of an acute MI would not be a preconditioning stimulus, but a perconditioning or a postconditioning stimulus, as the onset of ischaemia is unpredictable. Primary PCI is the gold standard, and the first study to examine RIC in this setting included 33 patients in each group, but was negative 95. Interestingly, in this study, while RIC did not provide additional benefit over the control group, a third group that received morphine in addition to RIC had less myocardial injury, suggesting a synergistic role for RIC with an opioid. However, in a well‐powered trial of 333 patients, RIC instituted pre‐hospital, before primary PCI, resulted in better myocardial salvage at 30 days compared with the control group 96. Over a mean follow‐up period of 3.8 years, the rates of MACCE were significantly lower in the RIC group, as was the all‐cause mortality 97. A recent meta‐analysis in the setting of cardiovascular interventions suggests benefit from RIC 98, and we have presented some key trials in Table 3. It is interesting to note that the clinical setting of an acute STEMI, with complete occlusion of the coronary arteries (TIMI 0 flow), closely resembles the pre‐clinical setting of ligation of the coronary arteries. Often, many of these patients are on no prior medication. In theory, this subgroup of patients with IHD should benefit most from the phenomenon of RIC. Certainly, smaller trials with strict inclusion criteria of TIMI 0 or 1 (total or near total occlusion) flow on coronary angiography before randomisation have shown benefit from RIC 99, 100.

**Table 3:** Key clinical trials of remote ischaemic conditioning (RIC) in patients with ST‐segment elevation myocardial infarction (STEMI) treated by percutaneous coronary intervention (PCI)

Study	n	Study comments	Results
Rentoukas et al. 95	96	Single‐centre unblinded study Three groups: control; RIC; and RIC with morphine RIC initiated before procedure and morphine administered before balloon inflation	No difference between control and RIC groups in peak Tn release ↓ TnI in RIC and morphine group vs control
Botker et al. 96	251	Single‐centre single‐blinded study RIC started in the ambulance on confirmation of STEMI Primary endpoint was myocardial salvage index determined by SPECT	↑ Myocardial salvage in the RIC group ↑ Left ventricular ejection fraction at 24 h in the RIC group but not at 30 days No difference in 30‐day MACCE
Crimi et al. 99	96	Multicentre single‐blinded study Remote ischaemic postconditioning in the lower limb Only TIMI 0 and 1 flow patients were randomly allocated (total or near total occlusion)	↓ CK‐MB AUC in the RIC group ↓ Myocardial oedema as determined on T2‐weighted CMR imaging in the RIC group Better ST segment resolution in the RIC group
Sloth et al. 97	251	Follow‐up of MACCE to study by Botker at al. Median 3.8 years follow‐up	↓ MACCE in RIC group compared to the control group
White et al. 100	190	Single‐centre single‐blinded study TIMI 0 flow (total occlusion) as a specific inclusion criteria Primary endpoint was MI size as measured with CMR	↓ Infarct size in the RIC group ↓ Myocardial oedema in the RIC group ↓ TnT in the RIC group ↑ Myocardial salvage in the RIC group

Tn, troponin; SPECT, single photon emission computed tomography; MACCE, major adverse cardiovascular and cerebral events; AUC, area under the curve; TIMI, Thrombolysis In Myocardial Infarction study; CMR, cardiac magnetic resonance.

## RIC and the role of anaesthetic agents

While the role of anaesthetic agents has been reviewed elsewhere in this journal 10, they have specific interactions with RIC protocols that will be detailed here. Numerous pre‐clinical studies have shown that volatile anaesthetic agents such as isoflurane are cardioprotective, and reduce myocardial IRI by activating similar pathways as IPC 101. Furthermore, it has been suggested in several clinical trials that inhalational anaesthetic agents may be beneficial over intravenous agents in patients undergoing cardiac surgery, although debate still exists 102, 103. In 2007, the American Heart Association, along with the Society of Cardiovascular Anesthesiologists, recommended that patients with a history of cardiac disease should preferably be anaesthetised with a volatile anaesthetic agent 104. However, a recent clinical trial failed to support this recommendation 105. In addition, there is animal evidence that propofol is protective against myocardial, renal and cerebral IRI 106, 107, 108 at dosages of 6 mg.kg^−1^.h^−1^, along the lines of the Bristol model of total intravenous anaesthesia 109. Furthermore, it has been shown that propofol in dosages of 120 μg.kg^−1^.min^−1^ (7.2 mg.kg^−1^.h^−1^) in cardiac surgery resulted in a lower troponin levels at 24 h compared with isoflurane alone or low‐dose propofol (60 μg.kg^−1^.min^−1^) 110. In fact, there is evidence to suggest that propofol acts as a free radical scavenger, which may be beneficial in the setting of cardiopulmonary bypass 111. In summary, while volatile anaesthetic agents are cardioprotective, it is possible that high‐dose propofol may also be cardioprotective.

Given this controversy, it is unclear whether anaesthetic agents enhance or block the effects of RIC. In a clinical trial involving children with congenital heart defects, anaesthesia was induced with sevoflurane and maintained with isoflurane, air and oxygen throughout the procedures. Although the exact dosages were not reported in this study, RIC proved to be cardioprotective 60. In the setting of adult cardiac surgery, Hausenloy et al. reported the maintenance of anaesthesia using a target‐controlled infusion of propofol to achieve a plasma concentration of 3–8 μg.ml^−1^ 64. Remote ischaemic conditioning proved protective in this setting as well. However, other groups have reported conflicting data. For instance, in a four‐arm randomised controlled trial, Kottenberg et al. randomly allocated patients to receive RIC or a sham protocol of RIC with either propofol or isoflurane anaesthesia. They reported protection in patients allocated to the isoflurane arm only, implying that propofol blocked the protective effects of RIC 75. This group has further proposed that RIC protected via activation of the signal transducer and activator of transcription 5 (STAT‐5) in humans, a known cardioprotective signal, while propofol prevented STAT‐5 activation 112, 113. On the other hand, in a trial by Zaugg and colleagues, patients allocated to RIC before cardiac surgery were not protected. All patients in this trial were anaesthetised with isoflurane, and the authors have suggested that isoflurane is already cardioprotective, and RIC does not add any further benefit 73, 114. Large trials such as ERICCA and RIPHeart may answer this question.

## Other forms of RIC

Remote ischaemic perconditioning (RIPerC) is a strategy where multiple cycles of ischaemia‐reperfusion are applied during the lethal ischaemia phase, but before reperfusion 115. It has been shown to activate similar pathways to remote ischaemic preconditioning (RIPreC). The clinical relevance of perconditioning is appreciable in patients admitted for an urgent PCI. For instance, during an acute MI, patients present to the hospital with ongoing ischaemia, and thus any stimulus applied would be RIPerC. In the cardiac surgical setting, RIPerC involves application of the remote stimulus after the aortic cross‐clamp has been applied. In a trial of 81 patients admitted for elective aortic valve replacement, patients who received RIPerC had significantly lower levels of troponin 5 min before removal of the cross‐clamp, and 30 min after removal compared with control and RIPreC. However, at 72 h, there was no difference in troponin levels, and the significance of RIPerC is yet to be defined in cardiac surgery 70.

Remote ischaemic postconditioning (RIPostC) involves the application of the remote ischaemic stimulus at the point of reperfusion. The mechanisms of RIPostC have not been fully elucidated, but there is evidence that it involves the survival activating factor enhancement (SAFE) pathway, rather than the conventional RISK pathway activated in RIPreC. Translation into the clinical arena came in the form of the trial by Hong and co‐workers. They randomly assigned 70 patients having off‐pump CABG to a combination of RIPreC and RIPostC in one group, and controls in the other. This resulted in a 48.7% reduction in peri‐operative injury, as determined by the 72‐h troponin area under the curve 116. However, in a larger trial by this group including 1280 patients, RIPreC and RIPostC together did not reduce a composite endpoint of major adverse outcomes, which included death, MI, arrhythmia, stroke, coma, renal failure or dysfunction, respiratory failure, cardiogenic shock, gastrointestinal complication and multi‐organ failure 76. Currently, De Hert's group is aiming to recruit 660 patients for a multicentre, randomised controlled trial comparing a control group with RIPreC, RIPostC and a combination of both. Their primary endpoint is the incidence of postoperative of atrial fibrillation, with secondary endpoints including length of ICU stay, hospital stay and MACCE 117. In the setting of acute MI, RIPostC resulted in a 20% reduction in periprocedural injury, but had no influence in elective PCI for stable or unstable angina 94, 99.

At this point, it is worth briefly mentioning the concept of delayed RIPostC. The basis of the concept lies in the supposition that reperfusion injury does not only occur at the point of reperfusion, but can continue for a while after reperfusion. Delayed RIPostC aims to influence protective pathways later in the reperfusion period. At the time of writing, there have been no clinical trials in humans, but pre‐clinical evidence exists that RIPostC may reduce injury in the brain and the heart 118, 119.

## Conclusion

Remote ischaemic conditioning is a novel strategy that has been shown to reduce myocardial reperfusion injury. While there have been both promising and disappointing results from trials, results from large multicentre randomised controlled trials such as ERICCA and RIPHeart are awaited. Anaesthetists are in the unique position of providing peri‐operative care to their patients, especially in cardiac and neurosurgery. If RIC proves to be beneficial, then anaesthetists may be at the forefront of providing peri‐operative organ protection to their patients. Even if RIC is not realised as a protective intervention, an understanding of mechanisms may lead to development of pharmacological agents that may be administered peri‐operatively by anaesthetists, in the hope of protecting vulnerable patients from myocardial injury following reperfusion. Over the years, research and development has reduced the risks of cardiac interventions and surgery dramatically. Remote ischaemic conditioning may help us reduce this even further.

## Competing interests

No external funding or competing interests declared.
